# Supplementary material for: Comparative single-cell atlases reveal injury-driven tubal epithelial regeneration as a window for ovarian carcinoma initiation
Source: bioRxiv. 2026 Mar 30:2026.03.26.714557. Preprint. [Version 1] doi: 10.64898/2026.03.26.714557 (PMC13060286; doi:10.64898/2026.03.26.714557)
Supplement: Supplement 1 [file media-1.pdf]

Supplementary Information

Comparative single-cell atlases reveal injury-driven tubal epithelial regeneration as a window for ovarian carcinoma initiation

Ralston et al.

Supplementary Figures

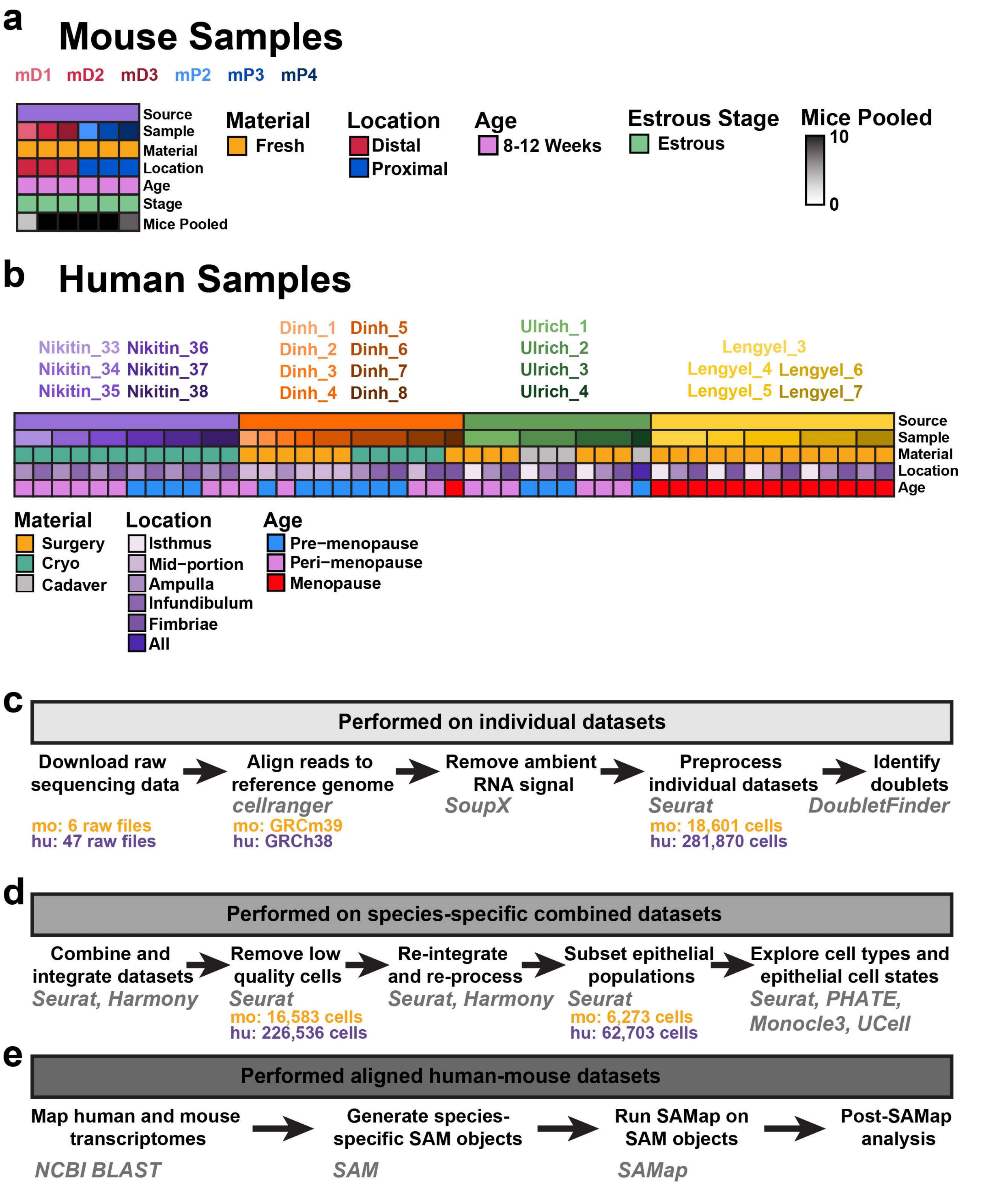

Supplementary Figure 1. Sample preparation and preprocessing. (**a**) Metadata visualization for the mouse scRNA-seq dataset. (**b**) Metadata visualization for the human scRNA-seq dataset. (**c-e**) Dataset preparation workflow beginning with individual dataset preparation (c), combined dataset batch correction and exploration (d), and multiple species alignment using SAMap.

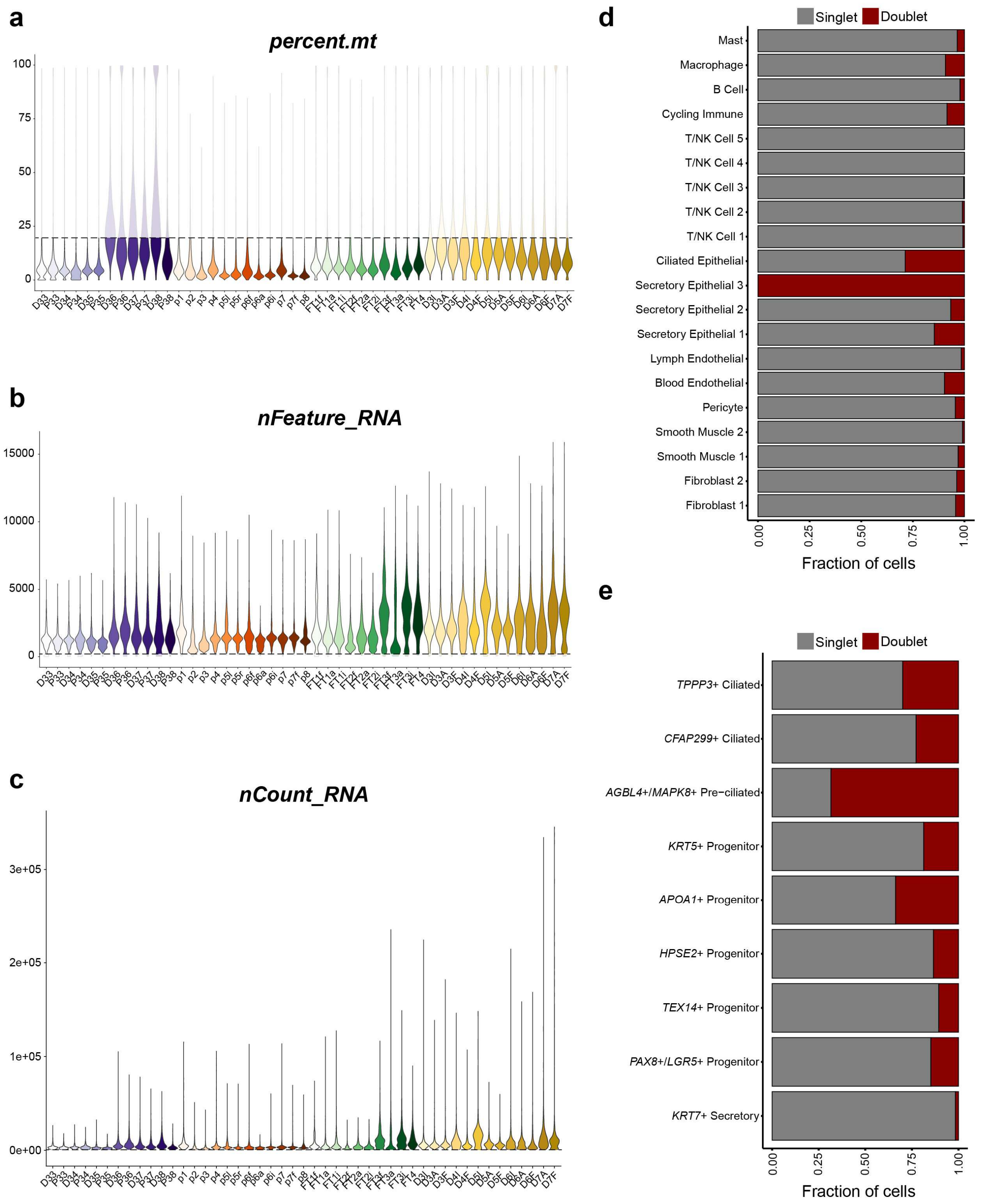

Supplementary Figure 2. Quality control for scRNA-seq dataset generation. (**a**) Mitochondria percentage was used to filtered cells with values greater than 20%. (**b**) RNA feature counts filtered out cells with values fewer than 200. (**c**) RNA counts filtered out cells with values fewer than 750. (**d-e**) Doublet detection using DoubletFinder for visualization of possible doublet populations in the whole human (d) and distal epithelial human (e) datasets.

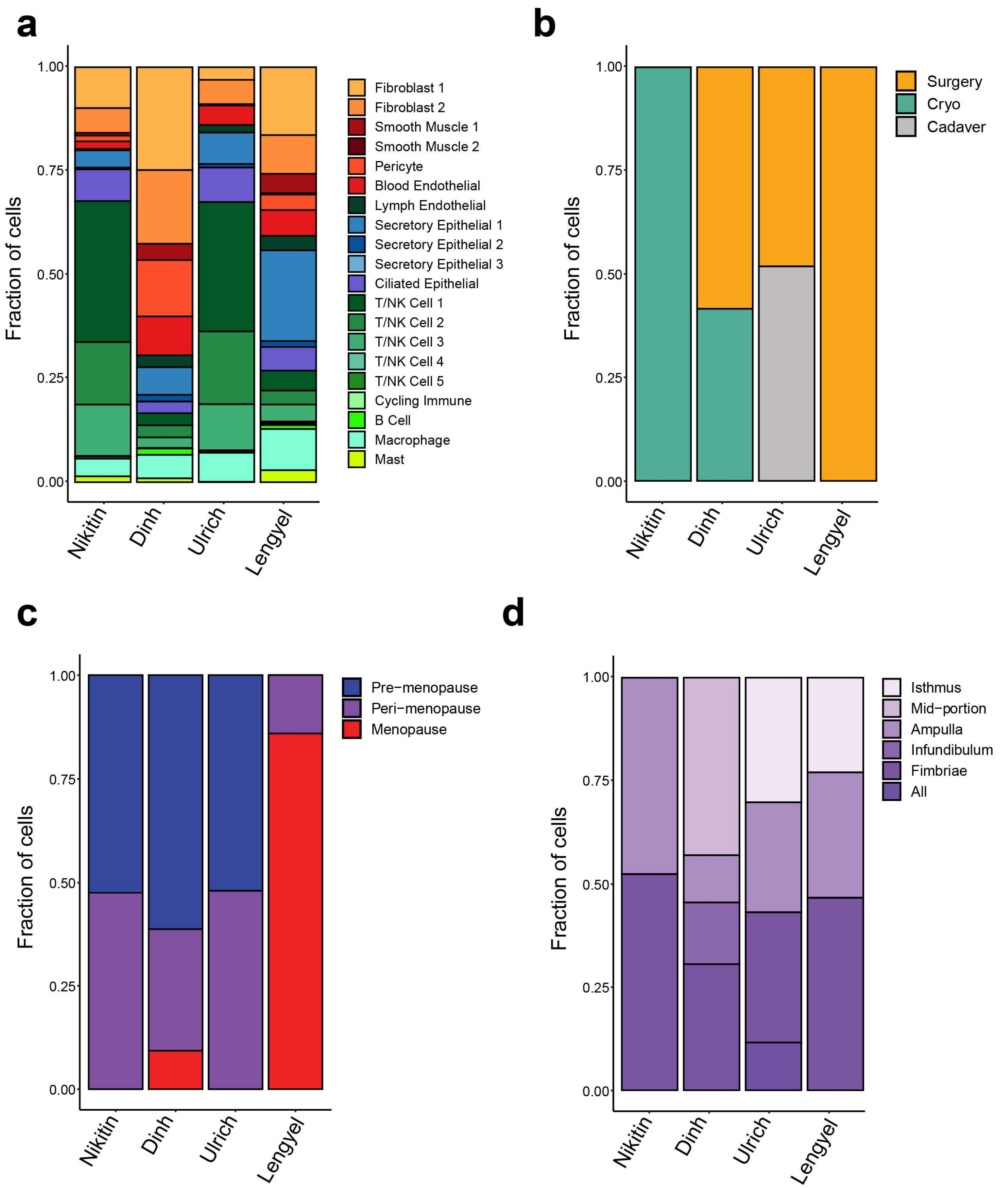

Supplementary Figure 3. Sample diversity based on source in human dataset. (**a**) Cell type distribution by source in which the sequencing files were obtained (**b**) Source differences in collected materials within the dataset. (**c**) Age group differences based on the source from which samples were derived. (**d**) Source-based distribution of regions collected for the human dataset.

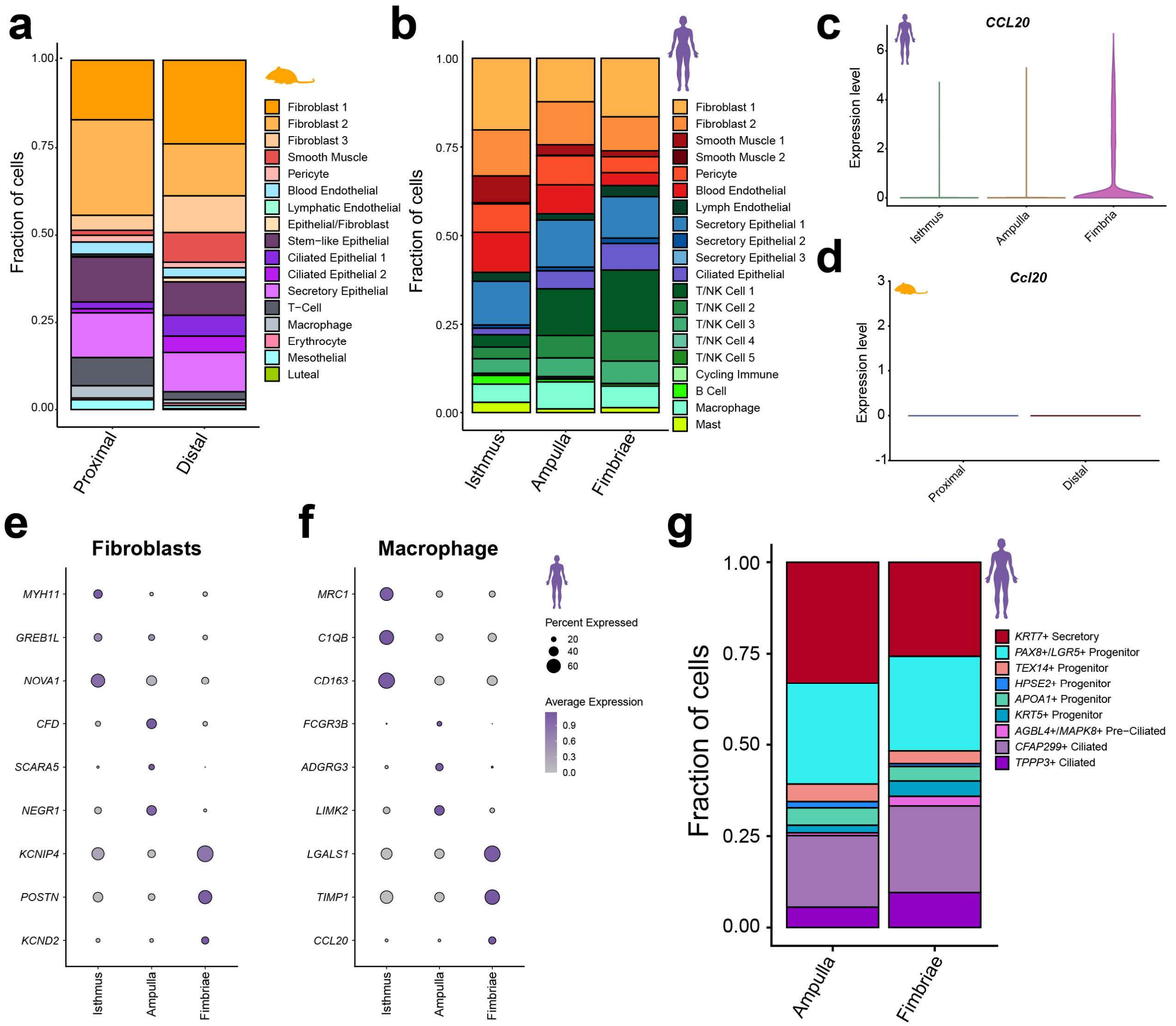

Supplementary Figure 4. Regionally-associated differences in human and mouse datasets. (**a**) Stacked bar chart showing cellular distribution for distal and proximal regions from the mouse uterine tube. (**b**) Stacked bar chart showing cellular distribution for isthmus, ampulla, and fimbria regions from the human uterine tube. (**c-d**) Violin plot expression of *CCL20* split across regions for macrophages from human (c) and mouse (d) datasets. (**e-f**) Top distinguishing markers exclusive to regions for human fibroblasts (e) and macrophages (f). (**g**) Stacked bar plot for cellular distribution among the ampulla and fimbria regions in human distal epithelial subsets.

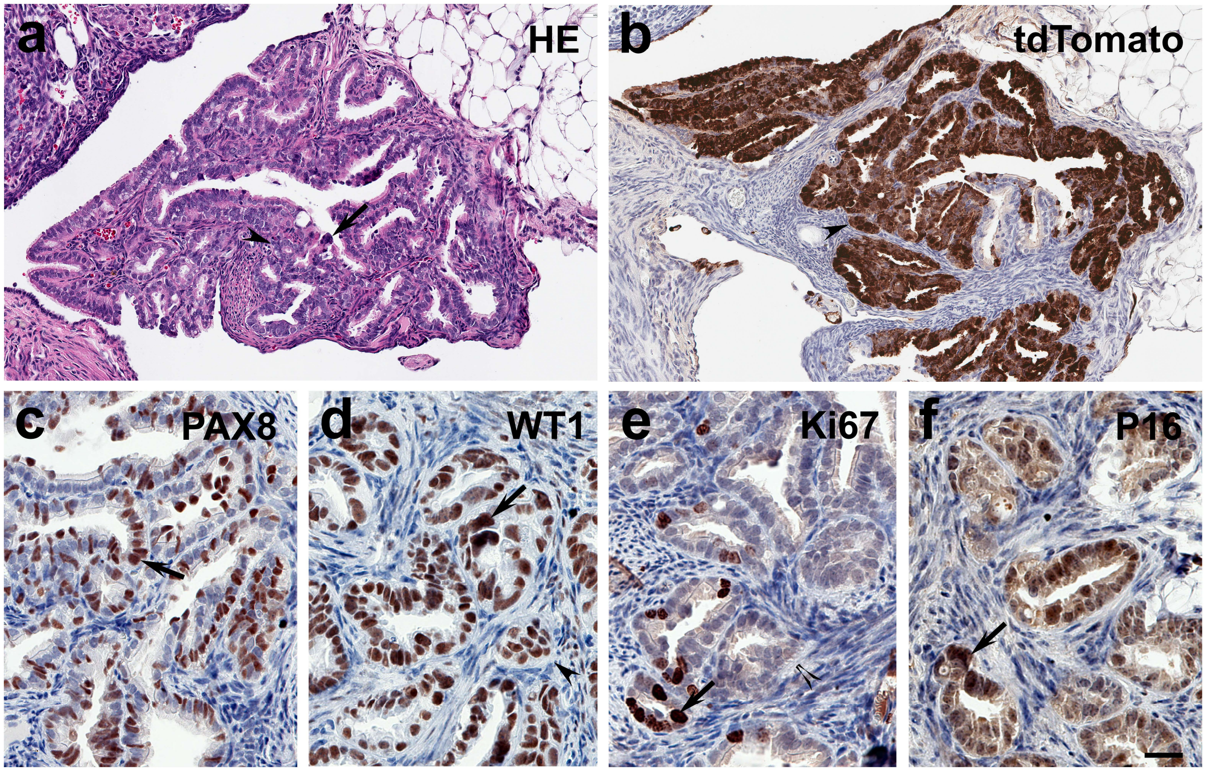

Supplementary Figure 5. Advanced neoplastic lesions in injured uterine tube of Krt5-CreERT *Trp53*$^{loxP/loxP}$ *Rb1*$^{loxP/loxP}$ Ai9 mice. (**a-f**) Lesions are marked by pronounced nuclear atypia (arrows) and stromal invasion (arrowheads), and expression of PAX8 (c), Wilms tumor 1 (WT1, d), Ki67 (e) and P16 (f). Hematoxylin and eosin (HE, a) staining, and immunostainings (brown color) for tdTomato, PAX8, WT1, Ki67 and P16 by Elite ABC method, hematoxylin counterstaining. Scale bar, 50 µm (a, b) and 25 µm (c -f).

Supplementary Tables

Supplementary Table 1. Neoplastic TE lesions in injured and non-injured contralateral uterine tubes after Cre-*LoxP* mediated inactivation of *Trp53* and *Rb1* in *Krt5*+ pre-ciliated cells

Injury	Yes	No
Cases, N	18	18
First detection (DPI)	26	120
End point (DPI)	208	208
Targeted cells (%)[#]	0.83±0.49	0.83±0.49
TE lesions (%)*	100	44
STIC	8	6
HGSC[&]	10	2

[#] According to tdTomato detection in Ai9 mice crossed to "Cre" mice

Mean ± SD

*TE lesions: injured vs non injured Fisher's exact P = 0.0003

[&]HGSC: Fisher's exact P = 0. 0116

Supplementary Table 2. List of genotyping primers

Mouse strain*	Gene detected	Primer name	Sequence (5' → 3')	PCR products
Rosa-loxP-stop-loxP-tdTomato/Ai9	Ai9	Ai9-1	AAGGGAGCTGCAGTGGAGTA	297bp-Wild type
		Ai9-2	CCGAAAATCTGTGGGAAGTC	196bp-Mutant
		Ai9-3	GGCATTAAAGCAGCGTATCC	
		Ai9-4	CTGTTCCTGTACGGCATGG	
K5-Cre-ERT2	*Cre*	Cre5'	GGACATGTTCAGGGATCGCCAGGC	269bp-Mutant
		Cre3'	GCATAACCAGTGAAACAGCATTGCT	
Rb1$^{loxP/loxP}$	*Rb1*	Rb212	CGAAAGGAAAGTCAGGGACATTGGG	295bp-Floxed
		Rb183'	GGAATTCCGGCGTGTGCCATCAATG	247bp-Wild type
		Rb19E	AGCTCTCAAGAGCTCAGACTCATGG	269bp-Knockout
Trp53$^{loxP/loxP}$	*Trp53*	P531F5'	GTGCCCTCCGTCCTTTTTCGCAATC	316bp-Floxed
		P5310F5'	GTTAAGGGGTATGAGGGACAAGGTA	163bp-Wild type
		P53102.3'	CCATGAGACAGGGTCTTGCTATTGT	198bp-Knockout

*See Methods for strain descriptions.

Supplementary Table 3. List of antibodies used for immunostaining

Antigen, conjugation	Antibody source, catalogue number	Clone	Host	Retrieval	Dilution
FoxJ1	Novus Biologicals, AF3619-SP	PC#	Goat	Citrate	1:800 (IF*)
Ki67	Thermo Fisher, 14-5698-82	SolA-15	Rat	Citrate	1:4000 (IHC&)
KRT7	Novus Biologicals, NBP2-44814	OV-TL12/30	Mouse	Citrate	1:200 (IF)
P16	Abcam, Ab241543	PABLO-33B	Rat	Citrate	1:500 (IHC)
p73	Abcam, Ab40658	EP436Y	Rabbit	Citrate	1:200 (IF)
PAX8	Proteintech, 10336-1-AP	PC	Rabbit	Citrate	1:4000 (IHC), 1:400 (IF)
RFP/tdTomato	Rockland Immunochemical, 600-401-379S	PC	Rabbit		1:4000 (IHC)
Periostin	Abcam, Ab14041	PC	Rabbit		1:3000 (IHC)
PLA2R1	Novus Biologicals, NBP1-84449	PC	Rabbit	Citrate	1:200 (IF)
ROBO1	Novus Biologicals, NB600-1254	PC	Rabbit	Citrate	1:200 (IF)
Wilm’s Tumor Protein 1	Abcam, Ab267377	EPR-23963	Rabbit	Citrate	1:500 (IHC)
Anti-goat IgG, Alexa Fluor 488	Invitrogen, A-11055	PC	Donkey		1:200
Anti-mouse IgG, Alexa Fluor 488	Thermo Fisher, A-11001	PC	Goat		1:200
Anti-rabbit IgG, biotinylated	Vector Labs, BA-1000-1.5	PC	Goat		1:200
Anti-rabbit IgG, Alexa Fluor 594	Thermo Fisher, A-21207	PC	Donkey		1:200
Anti-rat IgG, biotinylated	Vector Labs, BA-4000-1.5	PC	Rabbit		1:200

*IF, Immunofluorescence; #PC: Polyclonal; &IHC, Immunohistochemistry (ABC eliite method)
